# Mesenchymal Stem Cell-Based Therapy for Lysosomal Storage Diseases and Other Neurodegenerative Disorders

**DOI:** 10.3389/fphar.2022.859516

**Published:** 2022-03-02

**Authors:** Shaza S. Issa, Alisa A. Shaimardanova, Victor V. Valiullin, Albert A. Rizvanov, Valeriya V. Solovyeva

**Affiliations:** ^1^ Institute of Fundamental Medicine and Biology, Kazan Federal University, Kazan, Russia; ^2^ Faculty of Biology, Saint Petersburg State University, Saint Petersburg, Russia; ^3^ Department of Histology, Cytology and Embryology, Kazan State Medical University, Kazan, Russia

**Keywords:** lysosomal storage diseases, mesenchymal stem cells, neurodegenerative diseases, cell therapy, mesenchymal stem cell transplantation, clinical trials

## Abstract

Lysosomal storage diseases (LSDs) are a group of approximately 50 genetic disorders caused by mutations in genes coding enzymes that are involved in cell degradation and transferring lipids and other macromolecules. Accumulation of lipids and other macromolecules in lysosomes leads to the destruction of affected cells. Although the clinical manifestations of different LSDs vary greatly, more than half of LSDs have symptoms of central nervous system neurodegeneration, and within each disorder there is a considerable variation, ranging from severe, infantile-onset forms to attenuated adult-onset disease, sometimes with distinct clinical features. To date, treatment options for this group of diseases remain limited, which highlights the need for further development of innovative therapeutic approaches, that can not only improve the patients’ quality of life, but also provide full recovery for them. In many LSDs stem cell-based therapy showed promising results in preclinical researches. This review discusses using mesenchymal stem cells for different LSDs therapy and other neurodegenerative diseases and their possible limitations.

## Introduction

Lysosomal storage diseases (LSDs) are a group of approximately 50 genetic disorders caused by mutations in genes coding enzymes that are involved in cell degradation and transferring lipids and other macromolecules. Due to the accumulation of non-degraded macromolecules in lysosomes, LSDs present with different clinical symptoms (Maegawa 2019; Edelmann and Maegawa 2020). This group of diseases is rare in general but, altogether, their incidence ranges from 1 in 2,315 to 7,700 live-births (Meikle et al., 1999). Most LSDs affect the nervous system and are usually characterized by progressive neurodegeneration accompanied with delayed motor and cognitive functions (Maegawa 2019). All LSDs are inherited as autosomal recessive traits with the exception of Danon, Fabry and Hunter diseases (mucopolysaccharidosis type II), which are X-linked disorders (Wraith, 2002). Within each disorder there is a considerable variation, ranging from severe, infantile-onset forms to attenuated adult-onset disease, sometimes with distinct clinical features. In general, the earlier the onset, the more rapid the disease progression, variation can be also secondary to the residual activity of the metabolic pathway involved in each disease (Winchester et al., 2000; Kolodny 2003). LSDs can be classified according to the type of material that is stored. Major categories include sphingolipidoses, mucopolysaccharidoses, and glycogenosis (Bruce A. Fenderson 2009). Over the past several years the number of treatment approaches for patients with LSDs, like enzyme replacement therapy, substrate reduction therapy, hematopoietic stem cell transplantation and gene therapy has rapidly increased, but despite the remarkable advances, efficacy of most of these therapies is still limited, due to different obstacles (Beck 2018; Edward H.; Schuchman and Wassrstein, 2018; Solovyeva et al., 2018). The search for effective and safe therapy approaches for these diseases is of great interest. This review presents an overview of *in vivo* researches aimed at studying the effect of mesenchymal stem cell (MSCs) transplantation in LSDs and some neurodegenerative diseases.

## Stem Cell-Based Therapeutic Approaches for LSDs

The progressive nature of LSDs and limited efficacy of available treatments highlighted the need for further development of innovative therapeutic approaches, and for many LSDs stem cell-based therapy showed promising results in preclinical researches (Scruggs et al., 2012). MSCs are progenitor multipotent adult stem cells that can be derived from several tissues, like umbilical cord, bone marrow or fat tissue. MSCs can self-renew with the capacity to differentiate into multilineages: mesodermal (such as chondrocytes, osteocytes and adipocytes), ectodermal (neurocytes) and endodermal lineages (hepatocytes) making them a promising candidate for restoration of damaged tissue (Ullah et al., 2015; Chulpanova et al., 2021). Furthermore, MSCs are not only accessible for harvesting in clinical settings, but also can be easily grown in cultures, and are known for their ability to self-renew, express various lysosomal enzymes, modify immune reactions, be transduced by a variety of vectors and not to provoke an immune response (Scruggs et al., 2012; Ebrahim et al., 2020).

**FIGURE 1:**
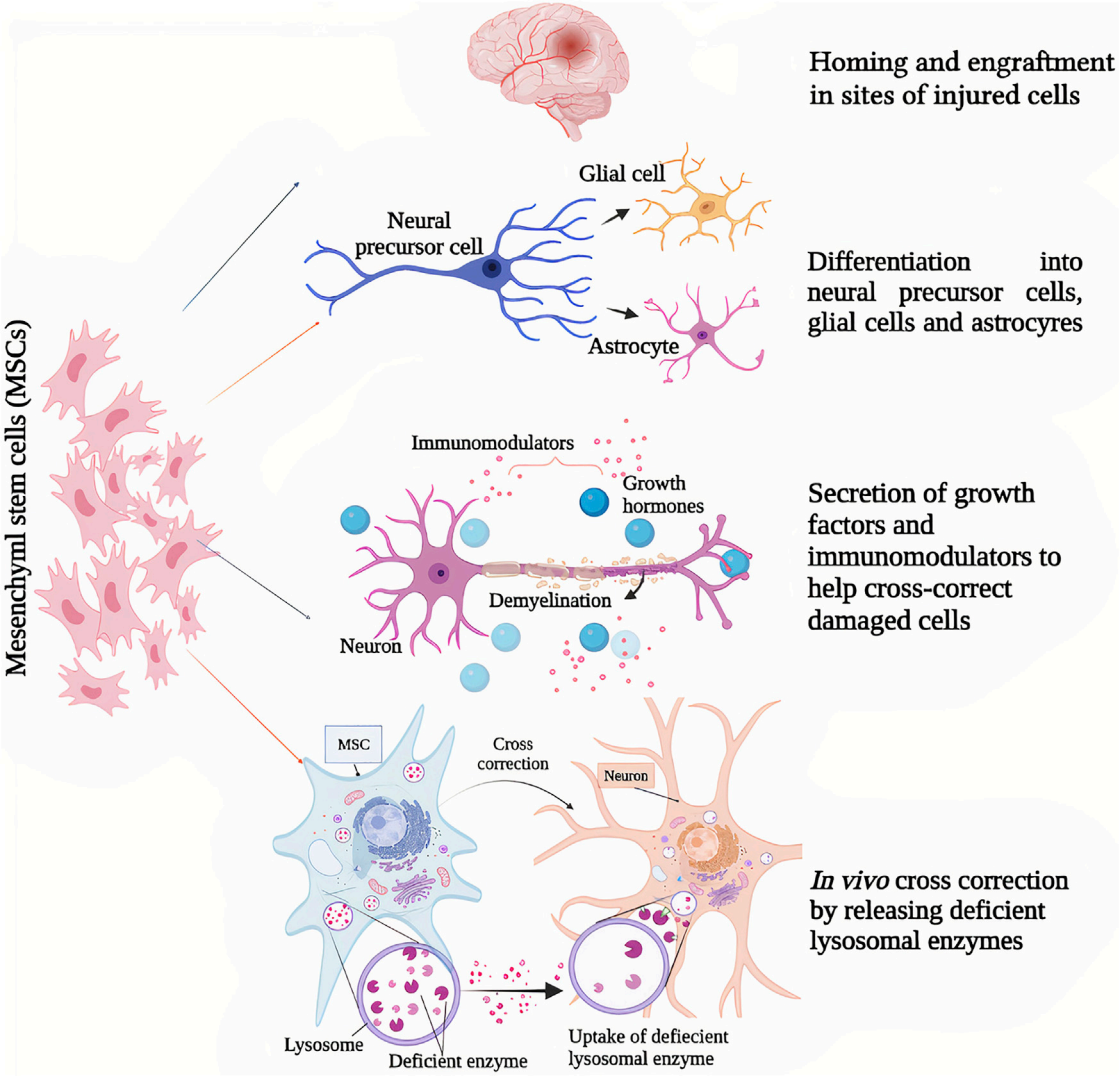
Mesenchymal stem cells’ (MSCs) mechanisms of action as potential therapeutic strategies for lysosomal storage diseases and other neurodegenerative diseases; (1) Homing and engraftment in sites of injured cells, (2) differentiation into neural precursor cells, glial cells and astrocytes, (3) secretion of growth factors and neuromodulators to help cross correct damaged cells, (4) *in vivo* cross correction by releasing deficient lysosomal enzymes.

Genetic modification of MSCs by transduction using recombinant virus vectors (as lentiviral or adeno-associated viral vectors) has allowed to use them for LSDs therapy to overexpress the inadequate or defective lysosomal enzyme in affected cells; or in some cases, even non-modified stem cells can produce sufficient amounts of endogenous lysosomal enzyme to make a cross-correction in LSD animal models (Hodges and Cheng 2006; Scruggs et al., 2012) (Figure 1). Table 1 provides details of existing *in vivo* and clinical studies on the efficacy of MSCs in LSDs and several neurodegenerative diseases.

**TABLE 1:** Therapies of LSDs and some neurodegenerative diseases using MSCs.

Disease	Study subject	MSCs dosage, route of administration and duration of therapy	Therapy results	References
Krabbe disease	Twitcher mouse model	1 × 10^5^ cells in 2 µl	Anatomical integration, with no tumor formation. Xenografts remained viable in both normal and twitcher mouse brain, exhibiting differentiation into astrocytes, neurons and oligodendrocytes	Croitoru-Lamoury et al. (2006)
		Intracerebral injection		
		35 days		
		20,000 cells in 1 μl per hemisphere	Moderate improvements in life expectancy and motor function. Decrease in markers of inflammation, macrophage infiltration, and microglial activation. Decelerated deterioration in the twitcher brain	Ripoll et al. (2011)
		Intraventricular injections		
		40 days		
MLD	Patients with MLD	2–10 × 10^6^ cells/kg	Well tolerance and no toxicity of MSCs infusion. Clear evidence of improvement of nerve conduction velocity in 4/6 patients	Koç et al. (1999)
		Intravenous injection		
		24 months		
	Patient with MLD	5 × 10^6^ CD34 ^+^ cells/kg followed by the infusion of 1 × 10^6^ cells/kg of MSCs	Stabilization of all previous neurological manifestations with no further deterioration. Aryl-Sulfatase-A levels increased to normal values 16 months after transplantation	Meuleman et al. (2008)
		40 months		
NP	BALB/c npc^nih^ (NP-C) mice	0.5 × 10^6^ cells in 3 µl	The transplanted cells infused with Purkinje neurons resulting in preventing their loss	Bae et al. (2005b)
		Intracerebral injection		
		4 weeks		
		1 × 10^5^ cells in 3 μl	Reduction of sphingosine accumulations in mutant Purkinje neurons. Transplanted cells also promoted survival of neurons and decreased neuronal apoptosis	Lee et al. (2010b)
		Intracerebral injection		
		1 week		
		Dose unidentified	Decreased inflammatory response as a result of suppression of astro/microglial activation by transplanted MSCs	Bae et al. (2005b)
		Intracerebral transplantation		
		4 weeks		
		1 × 10^6^ cells in 3 μl	Promoted survival of Purkinje neuron, inhibition of cholesterol accumulation or cerebellar apoptotic cell death	Lee et al. (2010b)
		Intracerebral injection		
		1 week		
MPS VII or Sly syndrome	MPS VII mice	1 × 10^4^ cells in 2 μl	Catabolism of GAGs accumulated in the cornea	Coulson-Thomas, Caterson, and Kao (2013)
		Intrastromal injection		
		Three different time frames for different groups of animals: 10, 6 and 2 weeks, respectively		
Fabry disease	Fabry ko mice	5 × 10^6^ cells	Reduction in lipid accumulation, delivering the deficient enzyme to different organs	Campbell et al. (2002)
		Bilateral injection into thigh muscles		
		4 weeks		
PD	Wistar rats	30 × 10^3^ in 3 μl	Improvement in motor functions, increasing in cells expressing tyrosine hydroxylase, which catalyzes the formation of L-dopa	Mostafavi et al. (2019)
		Intracerebral injection		
		8 weeks		
	Sprague-Dawley rats	23,000 cells in 8 μl and 1,80,000 cells in 8 μl in two groups of rats	Increase of neuroblast migration in the lesioned striatum, with multiple signs of MSCs potentials in promoting reparative mechanisms	Cova et al. (2010)
		Intracerebral transplantation		
		4 weeks		
AD	C57BL/6 mice	1 × 10^6^ cells in 2 μl	Significant reduction in Aβ levels following transplantation	Lee Jong Kil et al. (2009)
		Intracerebral injection		
		1 month		
	C57BL/6J mice	∼1×10^6^ cells in 500 µl	Improving spatial learning ability and memory function in the mice	Jiao et al. (2016)
		Intravenous injection		
		3 weeks		
	APP/PS1 double transgenic AD model mice	1 × 10^5^ cells in 3 μl	Modulation of microglial activation, alleviating the disease symptoms and cognitive regression	Ma et al. (2013)
		Intracerebral injection		
		25 days		
ALS	Patients with ALS	In phase 1 and 2 of the trial: Intramuscular (IM) injections at 24 separate sites (1 × 10^6^ cells/site) or intrathecal (IT) administration of 1 × 10^6^/kg cells	Indications of possible clinical improvement following transplantation, as 87% of the patients were found to be responders according to different clinical parameters like ALS Functional Rating Scale–Revised or forced vital capacity	Petrou et al. (2016)
		In phase 2a		
		Both IT and IM administration in 3 dosing cohorts		
		(Low dose: 1 × 10^6^cells/kg IT and 24 × 10^6^ cells IM; mid-dose		
		1.5 × 10^6^ cells/kg IT and 36 × 10^6^ cells IM; and high dose: 2 × 10^6^ cells/kg IT and 48 × 10^6^ cells IM)		
		3 months		
MS	Patients with MS	Single IV infusion of 1–2 × 10^6^/Kg body weight BMSCs (6 months follow up)	Well-tolerated cell infusion, no treatment-related serious adverse events, or evidence of disease activation were found after 6 months	Cohen et al. (2018)
		140 × 10^6^ UCBSCs intravenously over seven visits (20 × 10^6^ UCBSCs/day) separated by 1–4 days	UCBSCs transplantation was safe and feasible. No serious adverse events were recorded in patients. Improvement of symptoms was found 1 month after transplantation, and in some cases also after 1 year. Recorded improvements included expanded disability status scale (EDSS) scores, bladder, bowel, and sexual dysfunctions, improvement in walk times, general perspective of a positive health change and improved quality of life. 15 out of 18 patients showed inactive lesions in the brain and the cervical spinal cord after 1 year, and one patient showed close-to a complete brain plaques resolution	Riordan et al. (2018)
		Four infusions	No severe adverse events were recorded during 10-year follow-up, and combined administration was found to be safe and feasible, but also needed to be confirmed by future clinical trials in a larger cohort	Lu et al. (2020)
		Day 0: 40 ml of UCBSCs (4 × 10^7^ cells) infused intravenously		
		Days 7, 14 and 21, respectively: 20 ml UCBSCs (2 × 10^7^ cells) intravenously injected combined with 1 ml (2 × 10^7^cells) intrathecally injected		
HD	Transgenic HD mice of the R6/2 line	5 × 10^5^ cells in 2 µl	Significantly slowed striatal degeneration and behavioral impairment	Lee Soon-Tae et al. (2009)
		Intracerebral injection		
		12.5 weeks		
	C57/B6 and R6/2-J2 mice	3 × 10^6^ cells	Significant decrease of motor impairment. An increase in survival rate, as the transplanted MSCs were found to induce neural proliferation in the lesioned regions of the brain, and also the migration of microglia and neuroblasts to those regions	Lin et al. (2011)
		Unilateral intrastriatal injection		
		16 weeks		

## Specific LSDs and MSC Therapy

### Krabbe Disease

Krabbe disease (or globoid cell leukodystrophy; OMIM #245200) is an autosomal recessive neurodegenerative disorder that results from deficiency of the lysosomal enzyme galactosylceramidase. The disease has a prevalence of one in 100,000 births, and presents as demyelination of the central (CNS) and peripheral (PNS) nervous systems resulting from accumulation of partially metabolized galactocerebroside, with associated morbidity and mortality (Pastores 2009; Won et al., 2016; Pavuluri et al., 2017).

The twitcher mouse model is an authentic model of human Krabbe disease that developed through spontaneous mutation of the gene encoding galactosylceramidase at the Jackson Laboratory in 1976 (Kobayashi et al., 1980) this model presents biochemical and histopathological symptoms that are similar to the human form of Krabbe disease such as loss of oligodendrocytes or demyelination (Suzuki and Suzuki 1995; Ripoll et al., 2011). A study by Croitoru-Lamoury et al. (2006) showed that transplantation of MSCs in the twitcher mouse model of Krabbe disease led to anatomical integration, with no tumor formation, and according to the study; xenografts remained viable in both normal and twitcher mouse brain exhibiting differentiation into astrocytes, neurons and oligodendrocytes. Another study by Ripoll et al. (2011) evaluated the effect of intracerebroventricular administration of MSCs derived either from bone marrow (BMSCs) or adipose tissue (ADSCs) on Krabbe disease pathology. The obtained data in this study indicate that MSCs are potent inhibitors of inflammation processes associated with the disease progression, and that they may offer potential benefits as candidates for *in vivo* treatment approaches by reducing inflammation levels.

### Metachromatic Leukodystrophy (MLD)

MLD is an autosomal recessive demyelinating leukodystrophy belonging to LSDs, and caused by a deficiency of the lysosomal enzyme arylsulfatase A (ARSA), which is responsible for sulfatides degradation. Less commonly, the disease can be caused by deficiency of sphingolipid B activator protein (SapB), that activates ARSA enzyme and allows its binding to substrate (Shaimardanova et al., 2020). High concentrations of sulfatides accumulating in the white matter of nervous system result in demyelination of PNS and CNS, which causes symptoms like ataxia, seizures and impairment in both motor and cognitive functions (Biffi et al., 2008). Currently, there is no specific treatment for MLD, and its therapy is limited to palliative care and symptomatic management (Klein et al., 2006).

A study by Koç et al. (1999) proved that allogeneic transplantation of BMSCs could be effective for MLD treatment in humans. Although the clinical phenotypic correction was different from one patient to another based on the disease stage and patient’s age upon transplantation; but enzymatic activity was found to be higher than expected after transplantation. The study suggested also that MSCs represent candidates for corrective tissue therapy for such diseases as they can differentiate into several cell lineages, and their transplantation provides long term engraftment.

Later in 2002, as an improvement for this therapeutic approach using MSCs, Koç et al. (2002) suggested that intravenously infusion of culture-expanded MSCs can provide higher efficacy for MLD treatment. As a result, MSCs infusion was well tolerated, showed no toxicity, and despite the extended *ex vivo* culture using fetal calf serum-containing media, there have not been any hypersensitivity reactions. Moreover, four out of six MLD patients in the study showed clear evidence of improvement of nerve conduction velocity (NCV), that, according to the researchers, could reflect reversal in the neuropathology that leads to the loss of motor and sensory amplitudes. For further studies, scientists recommended targeted delivery of MSCs, co-infusion of donor MSCs simultaneously with hematopoietic transplantation or conducting multiple infusions to obtain more improved outcomes. And for better understating of NCV improvement mechanism, it was suggested to conduct further neurophysiologic studies before and after MSC therapy both in clinical and pre-clinical in animal models.

Another case was reported by Meuleman et al. (2008) of a patient who presented with the adult form of MLD and had developed progressive neurological manifestations over 1 year. Infusion of MSCs along with hematopoietic stem-cell transplantation (HSCT) were performed, and several checkups were done over the following 40 months. As a result, the patient showed stabilization of all previous neurological manifestations with no further deterioration, and Aryl-Sulfatase-A levels increased to normal values 16 months after transplantation.

### Niemann-Pick Diseases (NP)

The Niemann-Pick group of disorders is divided into four autosomal recessive types of the disease: A and B as acid sphingomyelinase deficiencies, and C and D as deficiencies of transport proteins (Vanier 2013). The first two types are classified as lipid storage diseases, that result in progressive accumulation of sphingomyelin either in visceral organs or in the brain (Vanier 1999), while C and D are characterized by cellular cholesterol trafficking defects resulting from mutations in the genes coding transport proteins NPC1 or NPC2, respectively (Vanier 2013).

A study by Bae et al. (2005b) investigated the relationship between neurodegeneration and MSCs fusion with Purkinje cells. According to the study, targeted transplantation of BMSCs prevented the loss of Purkinje neurons in the murine type of Niemann- Pick disease type C, as the transplanted cells infused with Purkinje neurons, which led to the suggestion of a combined therapy of gene delivery *via* transplanted MSCs that will serve as a vehicle to merge introduced genetic material with Purkinje cells. Later in 2010, Lee et al. (2010b) again described MSCs therapeutic effect mechanism on neuropathology of NP-C disease. It was found in the study, that cerebellum-targeted BMSCs transplantation led to reduction of sphingosine accumulations in mutant Purkinje neurons of murine Niemann-Pick C, transplanted cells also promoted survival of neurons and decreased neuronal apoptosis. Thereby, these obtained data describe how BMSCs have their therapeutic effect of on the neuropathology of NP-C disease.

Another factor that is thought to play a key role in neurodegenerative pathogenesis, is glial activation (Baudry et al., 2003), and according to a study by Bae et al. (2005a) MSCs are capable of decreasing inflammatory response in the murine form of NP diseases as they can suppress this astro/microglial activation which again makes MSCs a therapeutic tool candidate for NP disease and other degenerative disorders that have similar pathologies. Lee et al. (2010a) also supported this hypothesis in a study in 2010 using human umbilical cord blood derived MSCs (UCBSCs) transplantations instead of BMSCs in mice models of NP-C disease, which was also proved to have the ability to promote survival of Purkinje neuron, inhibit cholesterol accumulation or cerebellar apoptotic cell death and to be considered as a therapeutic candidate for neurodegenerative diseases.

### Mucopolysaccharidoses VII (MPS VII) or Sly Syndrome

MPS VII, also known as Sly syndrome, is an autosomal recessive disorder, belonging to the group of LSDs, and one of the rarest types of mucopolysaccharidosis, with an estimated incidence rate of 1 in 250,000 newborns around the world (Muenzer 2011; Montaño et al., 2016). The disease is caused by deficiency in enzyme’s β-glucuronidase activity, which is usually responsible for glycosaminoglycans (GAGs) degradation (Sly et al., 1973). GAGs accumulation leads later to cellular and organ dysfunction. The clinical presentation of this disease differs widely between patients, with some general manifestations like cognitive impairment, skeletal dysplasia, heart valve abnormalities corneal clouding, developmental delay and hepatosplenomegaly (Coulson-Thomas et al., 2013; Montaño et al., 2016). Therefore, as a result of the disease rareness and its wide spectrum of symptoms; to date there is still no effective treatment regimen for it.

A study by Coulson-Thomas et al. (2013) hypothesized that intrastromally injection of UCBSCs into cornea could help in GAGs degradation and treating corneal defects in the murine type of Sly syndrome. As a result of the study; transplanted MSCs enabled catabolizing the accumulated in cornea GAGs, suggesting that this approach could be applied to treat corneal defects in the human type of disease.

### Fabry Disease

An X-linked recessive disease and the only known X-linked sphingolipid storage disease, characterized by deficiency in the lysosomal enzyme α- Galactosidase A (α-Gal A), that leads to systemic accumulation of globotrioasylceramide (GL-3) and related glycosphingolipids, causing cardiac, cerebrovascular, and even end-stage renal disease depending on type of affected cells (endothelial, cardiac, renal etc.). Disease frequency ranges between 1/40,000 and 1/60,000 in men, while in heterozygous women severity differs as a result of uneven inactivation of chromosome X (Susan E. Campbell et al., 2002; Desnick et al., 2003; Masson et al., 2004).

In 1997 α-Galactosidase A deficient mice were developed as an animal model of Fabry disease that can have similar-to-humans symptoms. Later, a study by Susan E. Campbell et al. (2002) investigated the possibility to correct genetic defect in Fabry disease through bilateral injection of genetically modified human MSCs into thigh muscles of α-Gal A knock out mice. As a result, α-Gal A-hMSCs were able to reduce lipid accumulation in treated mice and to deliver the deficient enzyme to different organs.

## Using MSCs in the Treatment of Other Neurodegenerative Diseases

### Parkinson’s Disease (PD)

PD is a neurodegenerative disorder, that affects motor system with signs and symptoms that can be different from one patient to another. In general, it is characterized by tremors, bradykinesia, rigid muscles, loss of automatic movements and speech changes (Lang and Lozano 1998). The Key pathology of PD is loss of dopaminergic neurons, which can be linked to genetic or environmental factors. Currently, the main treatment for PD is drug therapy, which can improve only motor symptoms (Sulzer et al., 2017; Tang et al., 2020) as for nonmotor symptoms, they still cannot be improved by drugs.

A study by Mostafavi et al. (2019) evaluated using MSCs in rat model of PD, where green fluorescent protein (GFP)-labeled MSCs were transplanted in brains of rats (in the striatum specifically). The study results showed an improvement in motor functions of PD rats after MSCs transplantation and an increasing in cells expressing tyrosine hydroxylase, which catalyzes the formation of L-dopa.

A similar study by Cova et al. (2010) showed also that transplanting human MSCs in rat model of PD resulted in increase of neuroblast migration in the lesioned striatum of transplanted rats with multiple signs of MSCs potentials in promoting reparative mechanisms for PD treatment.

### Alzheimer’s Disease (AD)

AD is also a neurodegenerative disease and the most common type of dementia. The disease presents with progressive declining of behavioral and cognitive functions such as language, memory, comprehension, attention, judgment and reasoning (Kumar et al., 2021). AD’s pathogenesis has been linked to amyloid β plaques (Aβ) aggregations in specific areas of the human brain (Tiwari et al., 2019). To this date there is still no effective therapeutic or prophylactic approach for this disease.

A study by Lee Jong Kil et al. (2009) tested the effectiveness of using BMSCs as a therapeutic agent in an acutely induced AD animal model. The obtained data showed significant reduction in Aβ levels following intracerebral transplantation of BMSCs into the animals’ brains. Another study by Jiao et al. (2016) studied the potential therapeutic effect of intravenously injection of human placenta amniotic membrane-derived mesenchymal stem cells (hAMSCs) into AD transgenic mice. Following the injection, hAMSCs transplantation was found to be effective at improving spatial learning ability and memory function in the mice, which may be an indicator that hAMSCs could play a preventive or therapeutic role for AD.

Ma et al. (2013) also showed in their study that intracerebral transplantation of ADSCs in AD mice could modify microglial activation, alleviate the disease symptoms and cognitive regression, which again suggests using MSCs for AD therapy.

### Amyotrophic Lateral Sclerosis (ALS)

ALS is another neurodegenerative disease affecting the motor system. It starts with focal muscle weakness and stiffness, that progresses relentlessly to different muscles in different body regions, leaving the patient with a survival median of 3 years following the first symptom onset (Masrori et al., 2020). ALS occurrence has been linked to different factors including genetic factors, mitochondrial diseases and oxidative stress (Blasco et al., 2014). This disease is still incurable despite the high number of clinical trials for potential therapies, that were all proven to be negative (Cipollina et al., 2020).

A clinical trial by Petrou et al. (2016) investigated effectiveness and safety of intrathecal and intramuscular MSCs transplantation in ALS patients. Injected MSCs were induced to secrete neurotrophic factors. The trial results showed indications of possible clinical improvement following the transplantation, as 87% of the patients enrolled in the trials were found to be responders according to different clinical parameters like ALS Functional Rating Scale–Revised or forced vital capacity.

### Multiple Sclerosis (MS)

MS is a chronic, inflammatory disease of CNS, in which focal lymphocytic infiltration results in axonal damage and demyelination (Compston and Coles 2008). The disease is characterized in general by heterogeneity in symptoms, disease course, and outcomes due to the different involvement of motor, sensory, visual and autonomic systems. Optic neuritis, Uhthoff’s phenomenon (transient worsening of neurological symptoms with a rise in body temperature) and Lhermitte’s phenomenon (an electric shock-like condition, usually felt down the spine or limbs upon neck flexion) are characteristic of MS. (Doshi and Chataway 2016; Klineova and Lublin 2018).

MS is considered to be an autoimmune disease mediated by T helper (Th)1 and Th17 cells. Primary contact with an antigen leads to production of proinflammatory cytokines, which results in further Th cell up-regulation and eventually destruction of the blood–brain barrier (BBB), leading to Th cells migration into CNS (Klineova and Lublin 2018).

Therapeutic approaches of MS aim to treat acute attacks, improve symptoms and to stabilize, delay or slightly improve disability, which highlighted the need for new therapeutic approaches (Hauser and Cree 2020). To date, twenty-nine clinical trials worldwide were registered to evaluate the safety of MSCs transplantation in MS patients (Gugliandolo et al., 2020).

A phase 1 clinical trial, conducted by Cohen et al. (2018) proved the viability and safety of autologous BMSCs intravenous transplantation in MS patients. The trial’s results showed that cell infusion was well-tolerated, and at a 6-month follow-up there were no treatment-related severe or serious adverse events, or evidence of disease activation.

Riordan et al. (2018) also evaluated the efficacy of intravenous administration of UCBSCs for MS treatment in their open-label, single-arm, single-center phase 1/2 study. Obtained results showed that UCBSCs transplantation was safe and feasible. No serious adverse events were recorded in patients. Improvement of symptoms was found 1 month after transplantation, and in some cases also after 1 year. Recorded improvements included expanded disability status scale (EDSS) scores, bladder, bowel, and sexual dysfunctions, improvement in walk times, general perspective of a positive health change and improved quality of life. 15 out of 18 patients showed inactive lesions in the brain and the cervical spinal cord after 1 year, and one patient showed close-to a complete brain plaques resolution (Riordan et al., 2018; Gugliandolo et al., 2020).

Another trial conducted by Lu et al. (2020) evaluated the efficacy of combined intrathecal and intravenous administration of UCBSCs in MS patients. During 10-year follow-up, no severe adverse events were recorded, and combined administration was found to be safe and feasible, but also needed to be confirmed by future clinical trials in a larger cohort.

### Huntington’s Disease (HD)

HD is a late-onset autosomal dominant neurodegenerative disease characterized by motor impairment symptoms like involuntary choreatic movements, dystonia and impaired balance and posture, along with cognitive decline symptoms such as dementia, learning difficulties and psychiatric disturbances (Roos, 2010; Lo Furno et al., 2018). HD ends ultimately with death after 10–15 years of the first onset (Lo Furno et al., 2018). Currently available therapeutic drugs can only help improving the patient’s quality of life by relieving their symptoms.

A study by Lee Soon-Tae et al. (2009) studied the effect of ADSCs transplantation on HD pathology in rat model of the disease. Following intracerebral transplantation, striatal degeneration and behavioral impairment were found to be significantly slowed possibly because of the secreted factors by transplanted MSCs, according to researchers.

Another study by Lin et al. (2011) tested the effect of human BMSCs transplantation on HD mouse model. Obtained results showed a significant decrease of motor impairment and an increase in survival rate, as the transplanted MSCs were found to induce neural proliferation in lesioned regions of the brain, and also the migration of microglia and neuroblasts to those regions.

## Genetically Modified MSCs

In addition to the various potentials MSCs have in treatment or prevention of wide spectrum of diseases, MSCs can be modified to gain more potentials by increasing their survival, retention, migration capacities or growth factor production, principally through genetic engineering (García-Bernal et al., 2021). For example; in therapeutic approaches of neurodegenerative diseases, MSCs can be genetically engineered to overexpress specific neurotrophic factors like nerve growth factor (NGF) or glial cell line-derived neurotrophic factor (Lo Furno et al., 2018). Moradian et al. (2017) showed in their study in 2014 that engineering BMSCs to overexpress neurotrophin-3 (NT3) provided protection of nervous tissues and stimulated MSCs to differentiate (Damasceno et al., 2020). Li et al. (2008) also tested the effect of transplantation of BMSCs genetically engineered to overexpress NGF BMSCs into a rat model of AD. The study findings suggested that applying this approach may play an effective role in AD treatment, as the transplanted modified BMSCs were able to migrate to target sites, express NGF and significantly help improving memory and learning in the rats. In addition, modifying surface receptor expression in MSCs can enhance their homing and migration to the lesioned areas (Nowakowski et al., 2016), and increase their survival and viability rate. A study by Rizvanov et al. (2008) showed that genetically modified hUCBSCs to express human VEGF and mouse neural L1 cell adhesion molecule (L1CAM); were found to be able to successfully migrate into the nervous tissue of mouse model of ALS after transplantation, and survived for over 3 months. Another example of modifying MSCs to enhance their homing features was presented by Liao et al. (2016) in a study aimed to evaluate the targeted delivery of MSCs with triple PSGL1/SLeX/IL-10 engineering in mouse experimental autoimmune encephalomyelitis (EAE), a murine model for human MS (García-Bernal et al., 2021). Modified MSCs were found to have gained significantly enhanced homing features compared to unmodified MSCs. Moreover, IL-10-transfected MSCs showed substantial inhibitory activity on CD4 (+) T lymphocytes proliferation from EAE mice. As for *in vivo* treatment, modified MSCs evidently exhibited a superior therapeutic efficacy over unmodified ones in EAE mice, having significantly enhanced myelination and reduced lymphocytes infiltration into the white matter of the spinal cord.

MSCs can also be modified to express specific therapeutic genes using viral vectors, that can be introduced to cells by different techniques (Lo Furno et al., 2018). A study by Pollock et al. (2016) investigated the effect of injecting MSCs engineered by lentiviral transduction to overexpress Brain-derived neurotrophic factor (BDNF) into a mouse model of HD, as BDNF is known to be a lead candidate for HD treatment (Zuccato and Cattaneo 2007). Collected data showed significant reduction of atrophy and anxiety in mice after transplantation.

## Limitations of MSCs Cell Mediated Therapy for LSDs

Although clinical improvements have been documented in patients of different neurodegenerative diseases after using MSCs mediated therapeutic approaches; there still could be many limitations for applying these approaches. For example, in some cases patients need to undergo chronic immunosuppression upon transplantation, which restrains the utility of MSCs in therapy (Meng et al., 2003; Robinson et al., 2005). In addition, invasive intracranial or intrathecal delivery of MSCs may be required sometimes to overcome the blood brain barrier. And even after intracranial or intrathecal injection/infusion; migration capacity of cells may be limited, which makes it more difficult to achieve even distribution into the targeted area (Hodges and Cheng 2006).

Also, there are several factors that affect the therapeutic approach efficacy, like remaining enzymatic activity, nature of accumulated substrate or the age of patient receiving therapy, all these parametric variations result also in different outcomes after treatment with MSCs. And for some LSDs, overexpression of the deficient enzyme in the brain after MSCs therapy may be toxic or intolerable (Hodges and Cheng 2006; Selden et al., 2008; Scruggs et al., 2012). Moreover, according to Mukhamedshina et al. (2019) there is an important aspect that should be considered upon applying a therapeutic approach using MSCs, which is the donor related heterogeneity of MSCs expression, that could result in a wide variation of therapeutic efficacies. A study by Montzka et al. (2009) aimed to evaluate the capacity of human adult MSCs to differentiate into a neural lineage and to determine the degree of homogeneity between donor samples, found that different donor samples revealed different expression patterns, showing a substantial variation of marker expression. Having found these results, Montzka et al. (2009) also recommended that further studies should consider these inter-donor differences of MSCs prior to treatment.

Furthermore, the relatively small number of LSDs patients around the world limits the ability to design big world-wide clinical trials aimed at studying the best and safest therapeutic for humans.

## Conclusion

Despite the rareness of LSDs and limited efficacy of most of their current therapeutic approaches, as early onset and small patient numbers make it difficult for clinical trials to evaluate the effectiveness of various LSD therapy approaches, stem cell therapy in general and MSCs in particular represents promising improvement in the field of regenerative medicine thanks to their pluripotency and migration properties, especially if combined with gene therapy and other supplementary treatments. And although there is still a lot to do about safety and long-term efficacy, with research and efficient clinical studies we can gain more understanding to help improve lives of LSDs patients and overcome the current obstacles on the way of finding the best and safest treatments for them.
